# Internal Carotid Artery Aneurysm Mimicking Peritonsillar Abscess

**DOI:** 10.1155/2015/389298

**Published:** 2015-06-01

**Authors:** Jacek Brzost, Anna M. Cyran, Martyna Waniewska, Miroslaw J. Szczepanski

**Affiliations:** Department of Otorhinolaryngology, Faculty of Medicine and Dentistry, Medical University of Warsaw, Ulica Stepinska 19/25, 00-761 Warsaw, Poland

## Abstract

The extracranial internal carotid artery aneurysm (EICAA) is an uncommon arterial lesion. Patients typically present with neurologic symptoms resulting from impaired cerebral perfusion and compression symptoms of cranial nerves. Often EICAA presents as a pulsatile neck mass, which is otherwise asymptomatic. We present a case of an 84-year-old female, who was initially referred to the Emergency Department for Otolaryngology with suspected peritonsillar abscess. The patient had a history of recent upper airway infection and cardiovascular comorbidities, including hypertension and ischaemic stroke complicated by extensive neurologic deficits. Physical examination revealed a compact, nonpulsatile mass in the lateral parapharyngeal space and local erythema of the mucosa. Duplex Doppler Ultrasonography and Computed Tomography revealed an atherosclerotic aneurysm of the right internal carotid artery, measuring 63 × 55 × 88 mm, stretching from the skull base to the angle of the mandible.

## 1. Introduction

Extracranial internal carotid artery aneurysms (EICAA) are rare vascular lesions, most commonly caused by atherosclerosis. Manifestations are usually neurological and result from decreased perfusion of central nervous system; however, patients also present with symptoms of nerve and aerodigestive tract compression. Often the only sign is palpable neck mass [1, 2]. Herein we report case of a patient with EICAA, initially diagnosed with peritonsillar abscess, and provide a short review of relevant literature focusing on diagnostic procedure.

## 2. Case Report

**Figure 1:**
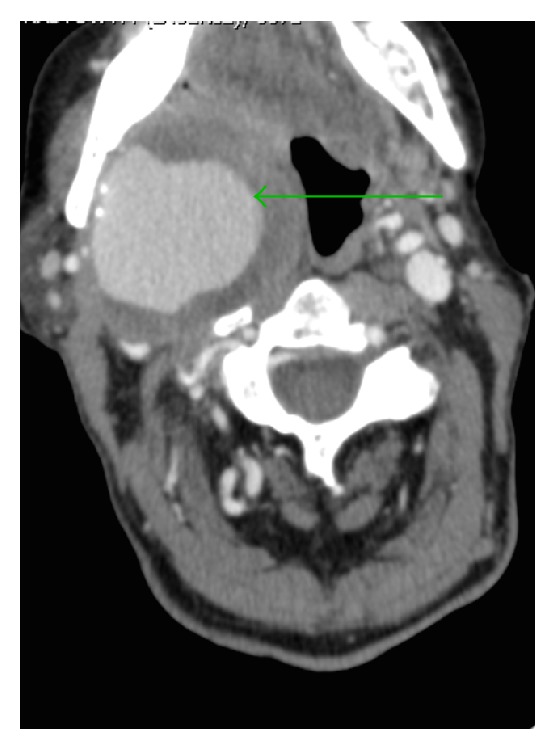
Computed Tomography showing extracranial aneurysm of right internal carotid artery (arrow).

**Figure 2:**
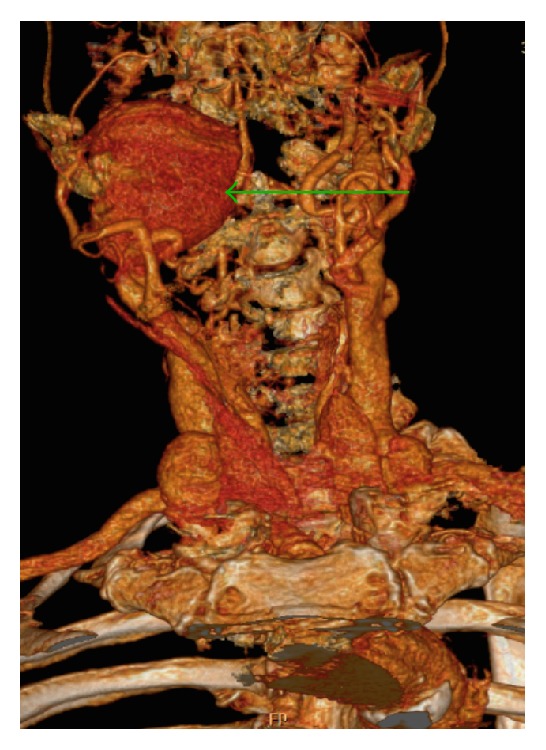
Digital reconstruction of Computed Tomography: extracranial aneurysm of right internal carotid artery (arrow).

An 84-year-old female with pain in the angle of the mandible and throat was referred to the Emergency Department for Otorhinolaryngology with suspected PTA. The patient had a history of cardiovascular diseases including hypertension and ischaemic stroke, complicated by left-sided hemiplegia, facial nerve palsy, hemispatial neglect, and dysarthria. History of trauma and previous vascular interventions was negative. The patient had dementia and was a nursing home resident, which impeded history taking. Physical examination revealed a compact, nonpulsatile mass in the right parapharyngeal space reaching the body midline. Mucous membrane was erythematous as a manifestation of pharyngitis sicca. Pyrexia, hoarseness of voice, and trismus were absent. Duplex Doppler Ultrasonography and subsequently performed Computed Tomography (CT) (Figure 1) with digital reconstruction (Figure 2) revealed a fusiform aneurysm of the right internal carotid artery. The aneurysm, with intraluminar thrombus and mural calcifications, measured 63 × 55 × 88 mm and stretched from the skull base to mandibular angle. The scan also showed a small saccular aneurysm of the left internal carotid artery. The patient underwent operative treatment in the Vascular Surgery Unit, comprising right internal carotid artery ligation. Operative technique was determined by proximity of distal end of the aneurysm to skull base. Postoperative course was uneventful.

## 3. Discussion

Internal carotid artery aneurysm is defined as a local increase in vessel diameter of >50%, compared to reference values for appropriate vessel segment. EICAA are uncommon arterial lesions; the estimated incidence is less than 1% of all arterial aneurysms and exceedingly rarely may be bilateral [1–3].

Reported etiology of EICAA varies greatly depending on population studied. According to the three largest studies, atherosclerosis is the leading cause of EICAA (43–91%). Other etiologies include fibromuscular dysplasia, aneurysm formation after surgical interventions, trauma, vasculitis, and infections [1, 2, 4]. Prevalence of causative factors has changed over the last 40 years and mycotic aneurysms are now sporadic; at the same time a higher incidence of dysplastic aneurysms is reported, due to improved screening of patients with carotid dissection. The incidence of traumatic EICAA remains at a relatively constant level of around 14% and justifies the low age average of patients with EICAA: 56 years of age [2].  Table 1 provides a review of etiology of EICAA.

Manifestations of EICAA depend on size and location and fall into three main categories: neurologic disorders caused by decreased perfusion of the central nervous system, symptoms resulting from compression of surrounding tissues, and hemorrhage. Neurologic disorders are most common and typically include focal symptoms such as strokes, transient ischaemic attacks (TIA), transient amaurosis, and anopsia. Less commonly described are Horner's syndrome, inarticulate speech, facial numbness, and retinal infarction [2, 3, 5, 6].

Large aneurysms displace tonsillar fossa medially and trigger compression symptoms, of which dysphagia is the most common and is caused by impaired function of the glossopharyngeal nerve (n. IX). Hoarseness of voice results from compression of recurrent pharyngeal and vagus nerves (n. X); local pain and discomfort are associated with malfunction of cervical branch of facial nerve (n. VII) [1, 7]. Further symptoms described include dizziness and tinnitus [2]. Hemorrhage has been described as a consequence of rupture of atherosclerotic and mycotic aneurysms [7, 8]. Often patients present with no symptoms other than a palpable mass at mandibular angle [3, 9].

Differential diagnosis of peritonsillar mass comprises carotid artery aneurysm and infectious and neoplastic etiologies. Patients with diabetes, the elderly, and those living in common quarters are at risk of developing infections and their complications, signs of which may be less apparent. The initially suspected PTA is the most common complication of acute tonsillitis. In a series of 15 EICAA, Szopinski et al. mention a patient with recurrent tonsillitis who sustained hemorrhage after an attempt of drainage of suspected PTA [10]. Further infectious causes to be considered are retropharyngeal cellulitis, infectious mononucleosis, mycobacterial cervical lymphadenitis, and Lemierre's syndrome (also known as postanginal sepsis). Neoplastic causes to be ruled out in these clinical circumstances include oropharyngeal tumour of the tonsil or very rare primary parapharyngeal space tumour. In a case series of 24 EICAA, an aneurysm was misdiagnosed as a neoplastic mass during otolaryngologic surgery, and the patient died of stroke, complicating emergency vascular surgical intervention. Other possible etiologies of peritonsillar mass include iatrogenic, posttraumatic, or spontaneous haematoma and ectopic thyroid gland, which may remain undiagnosed for years [5]. Duplex Doppler Ultrasonography is the diagnostic method of choice in suspected EICAA. Detailed evaluation of the anomaly is obtained with the use of CT angiography and Magnetic Resonance Imaging (MRI). In patients with neurologic symptoms, brain CT and MRI angiography may be employed to confirm or exclude ischaemia [4, 7, 11, 12]. In order to differentiate between dissection and aneurysm, flow signal inside the vessel may be recorded with use of transoral carotid ultrasonography [13]. Three-dimensional time-of-flight MRI angiography provides information about early stage vessel pathology and may be used for evaluation of treatment effects especially in patients with cardiovascular risk factors [11]. In patients with EICAA, further diagnostics should be performed in order to exclude concomitant cerebral and peripheral aneurysms, and chest CT angiography and Duplex Doppler Ultrasonography are recommended for this purpose [4, 9].

The first line of treatment in EICAA is open aneurysmectomy with end-to-end anastomosis or graft interposition [1, 4]. Other surgical modalities include recently described endovascular treatment and carotid ligation [10, 14, 15]. Operative technique depends on anatomic type of the aneurysm, as classified by Attigah et al. [1, 4]. Operation can be performed in general or regional anaesthesia [4, 15]. Conservative treatment carries a high risk of thromboembolic events and aneurysm rupture and thus is applicable in selected groups of patients only [2, 6].

## 4. Conclusion

Typical manifestations of EICAA are neurologic; however signs and symptoms observed most commonly by ENT specialists, such as neck mass, mass in the parapharyngeal space, dysphagia, dysphonia, and local pain, may be in the foreground. Thus a mass in the parapharyngeal space requires a careful differential diagnostic procedure, especially in patients with cardiovascular comorbidities. Palpation may reveal pulsation of the mass; however, lack of pulsation does not exclude presence of an aneurysm, which may be atherosclerotic with adherent thrombus.

## Figures and Tables

**Table 1:** Etiology of extracranial internal carotid artery aneurysms.

Study	Year	Number of aneurysms	Atherosclerosis	Dysplasia	Trauma	Infection	Postoperative aneurysms	Other
Zwolak et al. [6]	1984	24	24	Other etiologies excluded				
Faggioli et al. [5]	1996	24	9	12	1	0	2	0
Rosset et al. [3]	2000	25	9	12	3	0	Excluded	1
El Sabrout and Cooley [2]	2000	67	23	2	3	3	38	0
Szopinski et al. [10]	2005	15	5	0	6	0	2	2
Attigah et al. [1]	2009	64	42	3	0	1	7	10
Garg et al. [15]	2012	14	4	0	5	0	5	0
Radak et al. [4]	2014	84	77	2	0	0	5	0
